# Population Structure and Comparative Genome Hybridization of European Flor Yeast Reveal a Unique Group of *Saccharomyces cerevisiae* Strains with Few Gene Duplications in Their Genome

**DOI:** 10.1371/journal.pone.0108089

**Published:** 2014-10-01

**Authors:** Jean-Luc Legras, Claude Erny, Claudine Charpentier

**Affiliations:** 1 INRA, UMR1083 Science pour l’Œnologie, Montpellier, France; 2 Montpellier SupAgro, UMR1083 Science pour l’Œnologie, Montpellier, France; 3 Université Montpellier 1, UMR1083 Science pour l’Œnologie, Montpellier, France; 4 Université de Haute Alsace, Laboratoire Vigne Biotechnologies et Environnement, Colmar, France; 5 Institut Universitaire de la Vigne et du Vin, Université de Bourgogne, Dijon, France; University of Toronto, Canada

## Abstract

Wine biological aging is a wine making process used to produce specific beverages in several countries in Europe, including Spain, Italy, France, and Hungary. This process involves the formation of a velum at the surface of the wine. Here, we present the first large scale comparison of all European flor strains involved in this process. We inferred the population structure of these European flor strains from their microsatellite genotype diversity and analyzed their ploidy. We show that almost all of these flor strains belong to the same cluster and are diploid, except for a few Spanish strains. Comparison of the array hybridization profile of six flor strains originating from these four countries, with that of three wine strains did not reveal any large segmental amplification. Nonetheless, some genes, including *YKL221W/MCH2* and *YKL222C*, were amplified in the genome of four out of six flor strains. Finally, we correlated *ICR1* ncRNA and *FLO11* polymorphisms with flor yeast population structure, and associate the presence of wild type *ICR1* and a long Flo11p with thin velum formation in a cluster of Jura strains. These results provide new insight into the diversity of flor yeast and show that combinations of different adaptive changes can lead to an increase of hydrophobicity and affect velum formation.

## Introduction

Numerous fermented beverages have been developed all over the world during history. In addition to alcoholic fermentation, some beverages are obtained through a specific aging process called flor wine aging. During this process, which takes place only after the completion of alcoholic fermentation, a biofilm called velum is formed by yeast at the surface of the wine leading to the progressive oxidation of alcohol and remaining carbohydrates. This yeast oxidative metabolism generates many aromatic compounds (ethanal, sotolon, solerone…)[1]–[3], which give these wines their unique flavor.

Flor aging (or biological aging) is performed traditionally in several vineyards in Europe, including Hungary (Tokaj Hegyalja) to produce Szamorodni, Italy (Sardinia) to produce Vernaccia di Oristano, Spain (Jerez area) to produce Xeres, and France (Jura) to produce Vin Jaune. The apparition of the velum is generally spontaneous [4] but some French wine makers use selected flor starters. Flor yeast belong to the species *Saccharomyces cerevisiae* [5], and the population of flor yeast isolated from the velum of Sherry wines differs from the population of strains that perform alcoholic fermentation [5], [6]. These two populations are genetically isolated [7], as shown by the ITS1 region in Spanish and Jura flor strains, which have specific alleles of ITS1 caused by a 24 bp deletion [5] and a G insertion [8], respectively. Furthermore, various molecular techniques used to explore the diversity of flor yeast populations in several countries suggest a large genetic diversity [8]–[11].

Yeast strains adopt a specific lifestyle during flor aging, and adaptation to this ecological niche has long remained the focus of many investigations. Aneuploidies have been described [12], [13] as a major genetic feature of Spanish flor strains and were hypothesized to explain adaptation to flor aging. Indeed, yeast are able to adapt to stressful conditions due to the amplification of specific regions of their genome [14], [15]. The main adaptive feature of flor yeast is their ability to develop a velum on wine when sugars are depleted, which is an activity that is carried out only by some yeast strains [16]. The build-up of the biofilm is obtained by the aggregation of single cells, permitted by their high hydrophobicity. The high hydrophobicity of flor cells results from modifications of the lipid content and the activation of *FLO11* [17], which encodes a GPI anchored protein with a serine and threonine rich central region. Flor strains carry specific *FLO11* alleles that encode a protein with an expanded central hydrophobic core, which facilitates the adaptation of yeast cells to the velum environment [18], [19]. In addition, sherry flor strains have a deletion in the long noncoding RNA *ICR1* located upstream from *FLO11*. ICR1 functions as a switch that regulates the expression of *FLO11* and its disruption stimulates the expression of *FLO11* [18], [20].

Flor aging is encountered in highly distant vineyards, which raises the question of the relatedness and origin of these strains. The similar conditions faced by various strains in European vineyards implies that these strains share a similar genomic makeup and features of aneuploidy. In this paper, we compared flor yeast populations from Hungary (Tokaj), France (Jura), Italy (Sardinia) and Spain (Jerez). We used various molecular genetic techniques to investigate the genetic composition of these strains. The polymorphism of microsatellite markers allowed us to infer the structure of the flor yeast population. We measured the ploidy of strains and compared the genomes of several flor strains by CGH on array, which enabled us to detect aneuploidies specific to flor strains. Finally, we also examined polymorphisms within the promoter and protein central core region of *FLO11* and link these polymorphisms to the ability to grow on velum media.

## Material and Methods

### 1. Strains and growth conditions

The strains of this study originated from several laboratories in Spain, Hungary, Italy and France. They are described in detail in Table S1. The two first letters of Jura strains indicate the cellar from which each strain was isolated.

Yeast cells were cultivated in 10 ml of YPD medium (36 h, 28°C, 160 rpm). Velum growth was verified on Fornachon medium [21] (Yeast extract 1 g.l^−1^, (NH_4_)_2_SO_4_ 0.5 g.l^−1^, MgSO_4_ 1 g.l^−1^, CaCl_2_ 0.5 g.l^−1^, pH adjusted to 3.2 with HCl, autoclaved 35 minutes at 110°C, following which 4% (v/v) ethanol was added aseptically after cooling), after 8 days of incubation at 28°C.

### 2. Microsatellite typing and determination of population structure

*S. cerevisiae* microsatellite loci were amplified as described previously [22]. Genomic DNA was isolated by phenol/chloroform extraction, after cell grinding with glass beads, and isopropanol precipitation as described previously [23]. Allelic variation at 12 microsatellite loci was examined in 142 strains as described previously [22]. The chord distance Dc [24] matrix was calculated for each couple of strains with a laboratory-made program. The tree was obtained from the distance matrices with the Neighbor program of the Phylip 3.67 package, and drawn with MEGA5.22 [25]. The tree was rooted by the midpoint method. To assess the assignment of flor strains to a particular origin, InStruct [26] was used to evaluate the number of populations in the set of strains and a graphical display was obtained with R software version 2.15.1 [27].

### 3. Analysis of *FLO11* polymorphisms and cell hydrophobicity

The polymorphism of the length of Flo11p was measured from the amplification of *FLO11* alleles with a pair of primers located −53 bp in the 5′ of *FLO11* (Flo11IntFw CTCCCTCATCATGTTGTGGTTC), and +3126 bp in the terminal part of *FLO11* (Flo11IntRv AACGACGGTGGTTGAGACAA). ExTaq DNA polymerase (TaKaRa) was used to amplify this long DNA fragment. The PCR temperature program was 95°C for 5 min, followed by 30 cycles with an initial denaturation step of 95°C for 30 sec, annealing at 61°C for 30 sec, and elongation at 72°C for 6 min.

The presence of the 111 bp deletion in *ICR1* ncRNA was examined by the amplification of this region with the primer pair Flo11promFw CAGCCCCAGAGTATGTTCTCACAG and Flo11promRv AATCACCTTCTAAACGCTCGGA. This PCR was performed with regular MBI Fermentas Taq DNA polymerase. The PCR temperature program was 95°C for 5 min, followed by 30 cycles with a first denaturation step 95°C for 30 sec, annealing at 56°C for 45 sec, and elongation at 72°C for 1 min. The presence of the deletion was detected from the band size of the amplified fragment in gel electrophoresis.

For 5 strains (CAV21, LRJura, CECT11758, TR05CUB, T8CUB), the amplified fragment was sequenced with the same primers. These five sequences are available in GenBank under the accession number (HG965200–HG965204).

Cell hydrophobicity was evaluated following the procedure of Ishigami et al. [17], which relies on the measure of the partition of yeast cells between a buffer solution and an organic solvent. Yeasts strains were cultivated for 48 h with shaking in Fornachon’s media containing 4% ethanol, and then harvested, washed three times with water and suspended in 4 ml of McIlvaine buffer, pH 3.5. The cell population was adjusted to an optical density of approximately 0.5 at 660 nm (OD660). Four ml of this suspension was transferred to a test tube (15·150 mm) with a stopper. An equivalent volume of hexane was gently layered over the buffer. This test tube was vigorously vortexed for 5 min, with care taken to avoid emulsification. The OD660 of the initial and the residual buffer layers were measured, and the degree of hydrophobicity of the yeast cell surfaces (HD) was calculated from the equation:
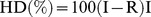
Where I and R are the OD660 of the initial and the residual layers, respectively.

### 4. CGH on array

Genomic DNA was labeled and hybridized against GeneChip Yeast Genome 2.0 Array from Affymetrix (Santa Clara, CA), which covers all *S. cerevisiae* S288C genes [28]. Labeled fragments were prepared from 200 to 500 ng of genomic DNA with the BioPrime DNA Labeling System (Invitrogen). The hybridization and detection steps were performed at the IGBMC Microarray and Sequencing Platform (Illkirch, France). Two arrays were used for each strain. Intensity data of perfect match probes were obtained with apt1.12.0 Affymetrix software, after RMA background subtraction and quantile normalization [29]. After filtering for probes with insufficient signal, the final number of probes used for the analysis was 38863. Signal intensities were scaled across arrays and log ratios were calculated using S288C as a reference. The log ratios were averaged by groups of three consecutive probes, to reduce probe to probe variation and facilitate analysis with DNAcopy. The best results were obtained after RMA background subtraction and quantile normalization of array data. Array Data were analyzed with the package DNAcopy [30] and R software version 2.15.1 [27]. A custom script was used to associate the mean log ratio calculated per chromosome segment with each ORF it contained. Gene clustering was performed with Cluster 3.0 [31], using a filter of 0.5 minimum difference in log ratio between all strains, and limiting missing data to six strains. Uncentered correlation and the centroid clustering were chosen as parameters, and dendrograms were drawn with TreeView. Gene ontology enrichment analysis was performed with Gene Codis 3.0 available at http://genecodis.cnb.csic.es/analysis [32].

The full data set has been deposited at the NCBI Gene Expression Omnibus (GEO) with GEO accession number (GSE55925) http://www.ncbi.nlm.nih.gov/geo/query/acc.cgi?acc=GSE55925.

### 5. Ploidies

For the analysis of cell DNA content, yeast cells were prepared in 96 well plates as described previously [33]. DNA content per cell was determined with an BD Accuri C6™ flow cytometer. However, Syber Green was used instead of Sytox Green because of the minimum variation observed with this fluorescent dye [34]. Both dyes give sharper peaks than propidium iodide, which has been used in most studies until now, and provide a more accurate evaluation of ploidy [34], [35]. By4741 and By4742 were used as haploid references and S288C and By4743 were used as diploid references.

## Results

### 1. Diversity of flor strains from various countries

**Figure 1:**
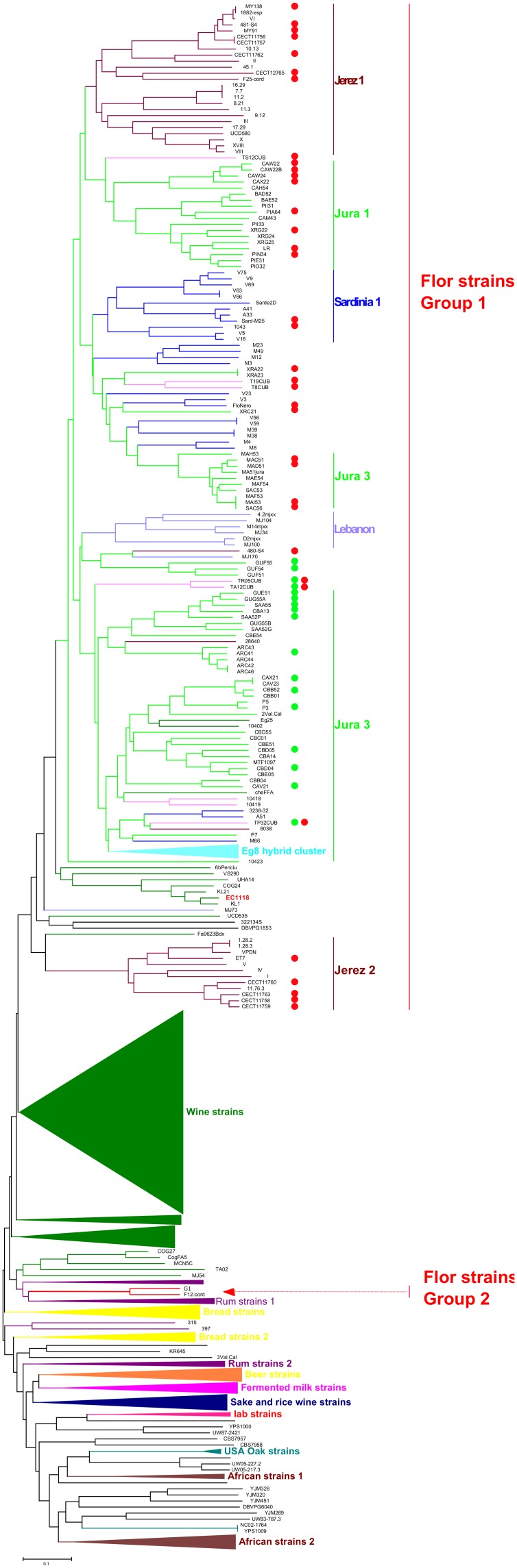
Neighbor joining tree presenting the diversity of flor strains evaluated at 12 microsatellite loci, in comparison with strains of other origins. The tree was built from the Dc chord distance and drawn with MEGA5.22. The wine cluster has been condensed due to its large size. Red dots indicate the presence of a 111 bp deletion in the *FLO11* promoter, and a green dot indicates that this deletion is missing.

We collected 142 flor stains from various countries. The 64 French strains from Jura were characterized previously by pulsed field gel electrophoresis and inter delta typing [8]. The other flor strains were provided by research groups from Spain (40 strains from the Jerez region and three strains from the Cordoba region), Italy (29 strains from Sardinia) and Hungary (6 strains from the Tokaj region). We evaluated the diversity of these 142 flor strains from polymorphisms detected at 12 microsatellite loci and were able to differentiate 131 genotypes. We compared these strains with 497 strains isolated from other sources (wine, palm wine, sake, oak bark) genotyped previously [22], [36] and 35 strains sequenced recently [37]. Flor strains clustered into one main group in a neighbor joining tree (Figure 1), with the exception of two Spanish flor strains isolated from Cordoba. Interestingly, subclusters formed inside the main group of flor strains according to geographical origin: three clusters of Jura strains, two clusters of sherry wine strains (Jerez 1 and 2), and one main cluster of Sardinian strains. In addition, Jura strains were grouped according to the cellar from which they were isolated. One Jura flor strain, MAA52, did not cluster with the other flor strains, and was thus considered as a wine strain.

**Figure 2:**
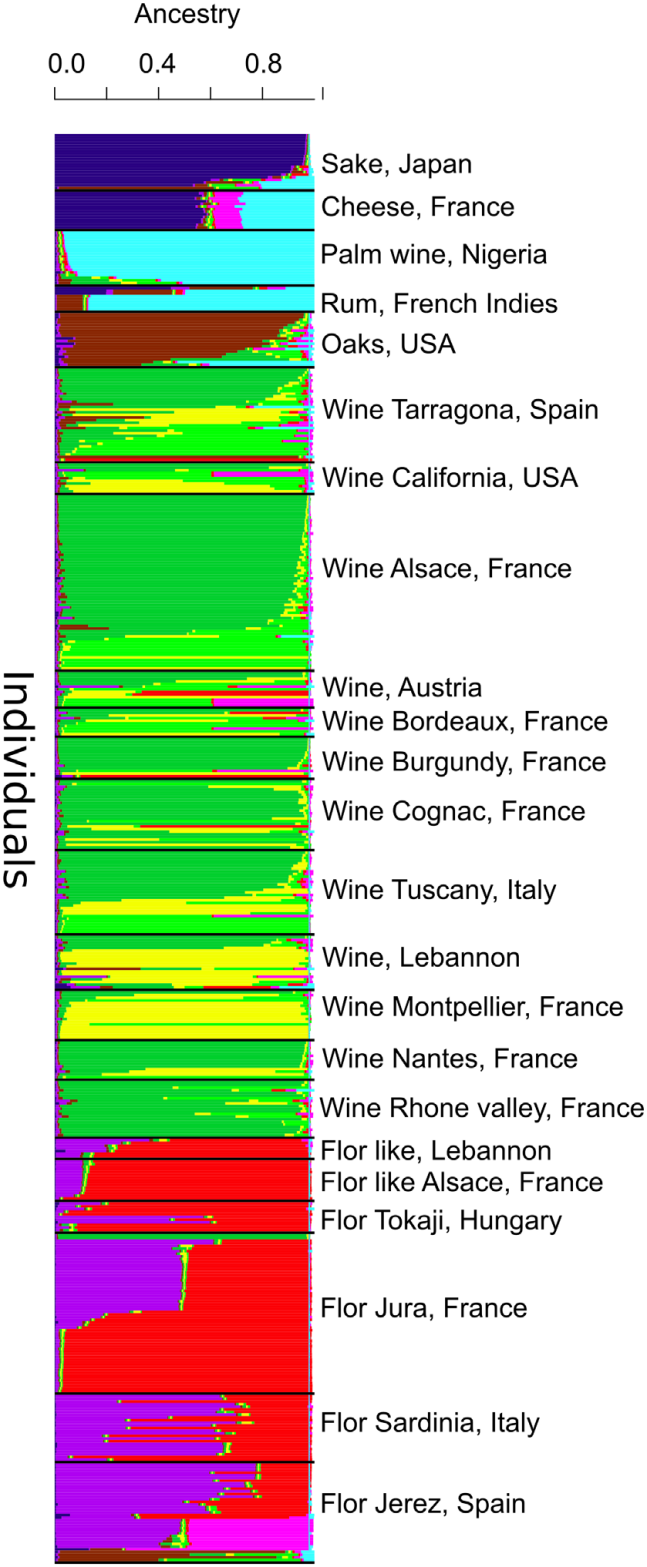
Clustering of flor strains with InStruct population structure inference software for K = 9 populations. Each color corresponds to one inferred ancestral group. The proportion of each colors gives the proportion of the corresponding ancestral genome in the genome of each strain. The name of the isolated population is shown at the top of each cluster.

To confirm the global structure observed from microsatellite typing, we used the software InStruct to detect population structure and assign the various flor strains to a particular origin. InStruct [26] is an alternative program to Structure [38] that takes into account partial self-fertilization and inbreeding; therefore, it is well suited for such an analysis because a high rate of inbreeding has been inferred from Fis values for yeast populations [22], [39], [40]. We selected groups of strains with sufficient members, reducing our strain set to 520, with the aim of limiting spurious clustering caused by an unbalanced effectives of the different origins. When evaluating the optimal number of ancestral lineages, DIC decreased sharply up to 9 and then continued to decrease up to 14, whereas a high variability appeared between 9 and 14 ancestral populations (FigureS1); therefore, K = 9 is the most probable partition inferred by InStruct. At K = 9, flor strains were assigned to two specific clusters (different from wine) (Figure 2). It is noteworthy that the separation of flor and wine clusters from strains of other origins already occurred at K = 3 (Figure S2).

**Figure 3:**
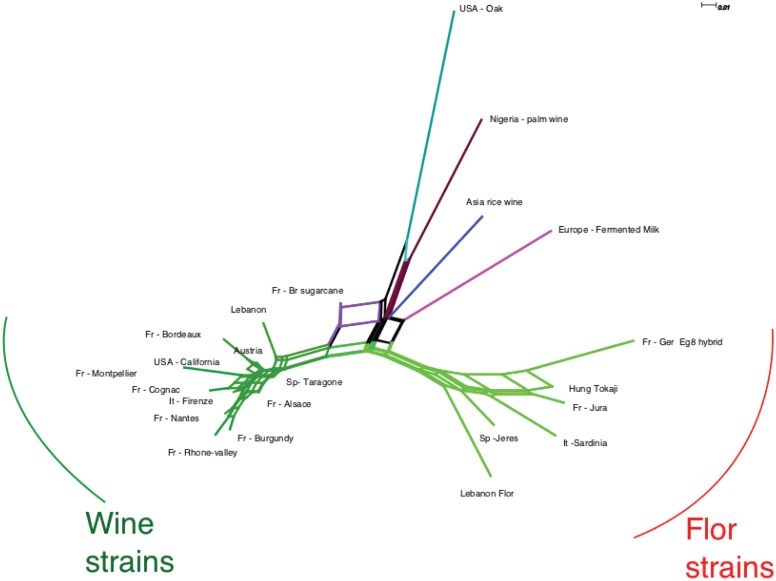
Neighbor net representing the differentiation between populations measured by Fst distance matrices.

The possible relationship between the different groups of flor strains can also be evaluated from the Fst genetic distance between each population. The neighbor-net network obtained with Splittree [41] from this distance matrix (Figure 3) separates clearly wine, flor and other strains into different groups, as suggested by InStruct. Interestingly, French and Hungarian flor populations are present at the end of the branches, whereas Lebanese and Spanish groups are the most basal.

### 2. Ploidies of flor strains

Flor strains have been described as aneuploid [13], [42] and variations in ploidy may explain differences in the properties of flor strains. We measured the DNA content per cell of 70 strains, which indicated that almost all strains were diploid, except three Spanish flor strains: F25 and Fino 1.28 that were triploid and Manzanilla X that was 2.6n (Table 1).

**Table 1:** Ploidy of flor strains from various countries (Spain, Italy, Hungary, and France) estimated from the DNA content measured in Flow cytometry.

Strain	Ploidy	CV %	Strain	Ploidy	CV %
**Spain**			**France**		
FINO 7.7	1.9	8.0	ARC42	2.0	4.3
FINO 11.3	2.1	7.8	ARC44	2.0	5.0
FINO 1.282	2.9	4.6	ARC46	2.0	6.3
Manzanilla-II	1.9	6.1	BAE52	2.1	8.0
Manzanilla-III	2.0	7.9	CAW24	2.1	5.0
Manzanilla-VI	2.0	9.0	CBA13	2.1	12.6
Manzanilla-VIII	2.1	9.2	CBB01	2.0	4.4
Manzanilla-X	2.6	4.2	CBB52	2.0	4.3
My138	1.9	6.6	CBD05	2.1	6.9
My91	1.9	6.9	CBD55	2.1	4.5
F25	2.9	5.0	GUF54	2.0	9.5
1682-S4	2.0	4.2	LRJura	2.0	5.4
CECT11761	2.0	4.7	MAC51	1.9	8.7
CECT11764	2.0	4.8	MAD51	2.1	5.7
G1	2.0	5.2	MAE53	2.1	5.2
**Italy**			MAE54	2.1	5.0
2D	2.0	8.3	MAF53	2.0	5.7
FloraNero	2.1	6.1	MAF54	2.0	5.0
A33	1.9	7.7	P3	2.0	7.1
A41	2.0	6.3	PIA64	2.1	5.2
A51	2.1	7.7	PII31	2.0	4.8
A9	2.0	8.4	PII33	2.0	6.5
M23	2.1	4.8	PIN34	2.0	6.5
M3	1.9	13.0	PIO32	2.0	5.5
M38	2.1	7.9	SAA52 g	2.0	7.2
M39	2.1	7.7	SAA55	2.0	6.1
M4	2.1	4.6	SAC56	2.1	6.7
M49	2.1	4.6	XRG25	2.1	5.5
M66	2.1	5.4			
M8	2.1	6.4	**Hungary**		
V23	2.2	7.7	T19CUB	2.0	6.3
V5sard	2.1	8.5	T8CUB	2.0	5.5
V63	2.0	5.6	TA12CUB	2.1	8.9
V75	2.0	4.9	TR5CUB	2.0	5.1
V80	2.0	4.9	TS12CUB	2.2	7.4
V9	2.0	5.0			

### 3. Comparative Genome Hybridization on array

Aneuploidy and gene amplification have been hypothesized as major sources of variation explaining adaptation to flor media [12]. We searched for a shared pattern of deletion or amplification specific to flor strains. We hybridized the genomic DNA of 11 strains of yeast to 2.0 Affymetrix chips using S288C as a reference. We tested six flor strains representing the four countries (LRJura from cluster “Jura 1”, P3 from cluster “Jura 3”, CECT11758 and My138 from cluster “Jerez 1”, TA12CUB from Hungary, and FloraNero from Sardinia) and four French wine strains (Eg25 and UHA13 isolated in Alsace, the haploid spore V5 from the champagne strain CIVC8130, and Eg8). The wine strain Eg8, a Saccharomyces *S. kudriavzevii hybrid, displays substantial aneuploidy [33] and was therefore chosen to verify our ability to detect large chromosomal imbalance. In addition, this strain has a microsatellite profile indicating that the *S. cerevisiae* moiety of its genome belongs to the flor yeast group.

**Figure 4:**
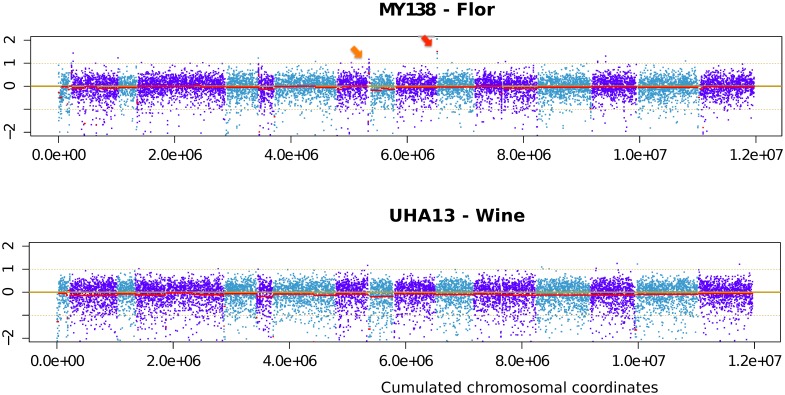
Karyoscope obtained with DNAcopy, showing variations in hybridization signal along the chromosome for flor strain My181 and wine strain UHA13. Chromosomes are colored in blue (uneven numbers) or dark blue (even numbers). Mean segment level estimated by DNAcopy is shown as a red line. The red arrow indicates the *YKL221W/MCH2* and *YKL222C* region, and the orange arrow indicates the *PHO12* and *IMD2* region.

A first analysis carried out with different normalization methods dedicated to Affymetrix arrays (RMA, GCRMA, MAS5) indicated that 1606, 834, and 218 probe sets, respectively varied significantly between strains after correction for multiple tests (adj. *p*. value <0.01). Although we were able to detect the main aneuploidies of Eg8, the high gene to gene variation in hybridization necessitated the use of a sliding window smoothing over three genes to reduce noise [33]. This explains why we used directly the signal of PM probes. We then chose to evaluate variation in copy number by detecting discontinuities of log ratios along the chromosomes with the Rpackage DNAcopy [30]. The hybridization patterns of each strain and discontinuities detected with DNA copy outputs for flor strain My138 and wine strain UHA13 are presented in Figure 4; other karyotypes are shown in Figure S3. As expected, aneuploidies were detected for *S. cerevisiae x S. kudriavzevii* hybrid Eg8 [33], and for the wine strain Eg25, isolated in Alsace. The microsatellite profile of Eg25 suggests that it is also present in the flor cluster. This strain has three main aneuploidies: two at chromosome III (there is only one copy of *YCL073C* to *YCL036W*, encompassing HMLALPHA1, but three copies of *YCR028W* to *YCR102W*, encompassing HMRA1 and 2) and one at chromosome XVI (from *YPL278C* to *YPL094C*). The anomaly of chromosome XVI involves a trisomy of the left arm of the chromosome starting at *YPL094C*, close to the promoter of *SSU1 (YPL092W)* [43].

In contrast with these aneuploid strains, we did not find substantial aneuploidy in the six flor strains tested. A low hybridization signal for chromosome I suggested the presence of only one copy in the CECT11758 strain, making it the only flor strain with a typical aneuploidy. Interestingly, a low hybridization signal at each subtelomeric region leading to an inverted U hybridization profile occurred in three of the six flor strains tested (TA12CUB, P3, and FloraNero), suggesting divergent alleles or missing genes in these regions.

**Figure 5:**
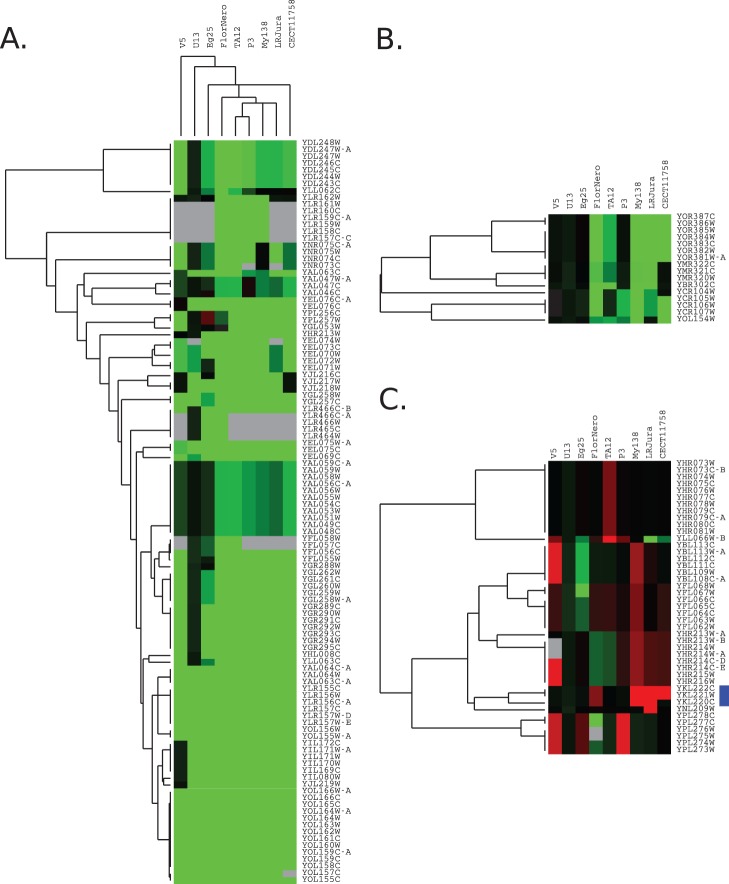
Hierarchical clustering of array CGH profile. Main clusters of gene with inter strain variability. A. Genes with a low hybridization signal for most strains. B. Cluster of genes with a low hybridization signal specifically for flor strains. C. Clusters of genes potentially amplified (Log ratio>0.3) in comparison with S288C.

For all strains, the hybridization signal of several genes was lower than that of the reference strain S288C. This suggests either the existence of divergent genes or genes with a low number of copies. We defined three thresholds to differentiate regions with zero, one, two or three copies: −1, −0.38 and +0.3, taking into account the average values observed for aneuploidies of CECT1158, Eg25 and Eg8 strains (Chromosome I of CECT11758, chromosome III and XVI of Eg25 and chromosomes IV, V, VIII and XVI of Eg8) and the dispersions around this average ratio. Accordingly, we divided regions with a low hybridization signal into two categories according to their log ratio: log ratio between −0.38 and −1, indicating one copy, and regions with hybridization signal lower than −1, indicating no copies. The gene lists corresponding to these thresholds are shown in Table S2, and the results of the comparison of these lists is shown in Table S3. We performed a clustering of the log ratio profiles, which revealed three main clusters (Figure 5). Interestingly, the global clustering separates flor and wine strains, suggesting that flor strains share copy number variation (CNV) profiles.

Cluster A (Figure 5A) contains 109 genes with a low hybridization signal. Twenty-four genes were apparently missing in all strains (flor and wine), and another 24 were missing in eight out of the nine strains. Among these genes, the cluster containing *ASP3–1/YLR155C* and *YLR157W–E* that was missing in all strains, and the neighboring genes *ASP3–3/YLR158C, ASP3–4/YLR160C, YLR161W,* and *YLR162W* that were missing in the genome of four flor strains, were detected previously in wine isolates [44], [45]. A second block of 15 genes from *HPF1*/YOL155C to *AAD15*/*YOL166C* on the left subtelomeric zone of chromosome XV, including the ferric enterobactin transporter *ENB1*, and the hexose transporter *HXT11/YOL156W*, is also missing among wine strains [44], [45]. We also observed the loss of a block of seven genes on chromosome VII, including *MAL13/YGR288W* and *MAL11/YGR289C* which are involved in maltose metabolism, and another cluster located on the left end of chromosome X containing an isomaltose α-glucosidase *IMA5*/*YJL216C*, three other genes *REE1/YJL217W*, *YJL218W* and the hexose transporters *HXT9/YJL219W*. Two other subtelomeric regions detected in wine strains analyzed previously by other groups [44], [45] were missing: a region containing eight genes from *HXT13/YEL069C* to *YEL075W–A* and another containing five genes from *IMA3/YIL172W* to *YIL169C*. *CUP1–2/YHR54C* was missing in all flor strains (except FloraNero), and in the wine strain Eg25, and *CUP1–1/YHR053C* was present in only two of the six flor strains.

In addition to the set of genes showing low hybridization, some genes showed moderately low hybridization, as exemplified by two subtelomeric clusters. The first cluster includes *AAD4*/*YDL243C, HXT15/YDL245C, MPH2/YDL247W, SOR2/YDL246C, COS7/YDL248W, YDL247W–A,* which was only present in wine strain UHA13. Interestingly this region was noted as giving a high amplification signal for wine strains, thus differentiating wine strains from strains of another origin [46] A second cluster, *PEX22/YAL055W, GPB2/YAL056W, YAL056C–A, CNE1/YAL058W, ECM1/YAL059W, YAL059C–A, BDH1/YAL060W, BDH2/YAL061W* and, *GDH3/YAL062W,* showed moderately low hybridization for five out of six strains, whereas other wine strains presented a hybridization log ratio close to 0 for this region.

Cluster B (Figure 5B) contains genes that are either missing or present with a low copy number in the genome of flor strains. One cluster of seven genes is located close to the right end of chromosome XV and contains several genes involved in iron import into the cell. These include the siderophore retaining proteins *FIT2/YOR382C* and *FIT3/YOR383C*, the siderophore Ferric reductase *FRE5/YOR384*W, and genes with other functions: *YOR381W–A*, *YOR385*W, *PHR1*/*YOR386*W, and *YOR387C*. A second subtelomeric cluster contains *PAU3*/*YCR104W*, ADH7/*YCR105W*, and RDS1/*YCR106W*, AAD3/*YCR107W* and a third cluster located at the right end of chromosome XIII contains *YMR320W*, *YM321C* and *SNO4/YMR322C.* The low hybridization of genes from the first and second clusters was detected previously by Caretto et al. [45] in the genome of two clinical isolates.

The presence of several clusters with low hybridization signals in subtelomeric regions is puzzling. These clusters explain the typical “inverted U” observed in Figure 4 (and in Figure S3) for several chromosomes of three flor strains: TA12CUB, P3 and in particular, FloraNero.

Few functional categories were associated with these genes. Genes involved in maltose metabolism were significantly affected (GO:0000023: maltose metabolic process, *p*. value = 4.6 e^−6^), as well as other hexose transporters. Nine of these genes encode proteins that are located in plasma membrane (GO:0016021: integral to membrane, *p*. value  = 0.0062), including several involved in iron uptake.

In addition to gene loss, gene amplification may also drive adaptation in response to a selective constraint [47]. We analyzed genes showing a higher hybridization signal for tested strains than for the reference control (Figure 5C); however, we found that only three genes were amplified in some, but not all, flor strains. These included *MCH2/YKL221W* and *YKL222W* that were amplified in LRJura, My138, CECT11758 and FloraNero strains (red arrows in Figure 4 and blue square in Figure 5), and *FRE2/YKL220W* in LRJura, My138, and CECT11758. The hybridization signal indicated that these genes were present in four copies in My138, LRJura and CECT11758, and three copies in FloraNero. Five other genes, *YAR064W, YAR068W, YHR214W, PHO12, IMD2* showed a high hybridization signal in three flor strains (LRJura, My138, and CECT11758). A second cluster of genes including *YHR213W–A*, *YHR213W–B*, *YHR214W–A*, *YHR214W, YHR214C–D*, *YHR214C–E*, *PHO12/YHR215W, IMD2/YHR216W* (red arrow) showed a high hybridization signal in the V5 strain, which was described previously for the wine strains EC1118 and ICV D254 [45]. The average log ratio in this region suggests three copies for LRJura, My138, and four copies for V5. Another cluster of six genes, located at the extremity of the left arm of chromosome XVI, containing the genes *SAM4/YPL273W, SAM3/YPL274C, FDH2/(YPL275W, YPL276W), YPL277C, YPL278C* was amplified in the genome of three strains (wine and flor): P3, Eg25 and V5. Dunn et al. observed previously the amplification of this region in several wine strains [44]. Another subtelomeric cluster encompassing *YFL062C* to *YFL068W* presented a high hybridization signal in strain My138 (log ratio 0.48). This was also the case for strains CECT11758, TA12CUB, FloraNero and V5; however, the log ratio for these strains was below 0.3 (between 0.23 to 0.24), thus the genes were not considered as amplified. Interestingly, except for the cluster containing *YHR073W* to *YHR081W* that was amplified only in TA12CUB, all the clusters containing amplified genes were subtelomeric.

### 4. Variability in velum production and *FLO11* polymorphism

**Figure 6:**
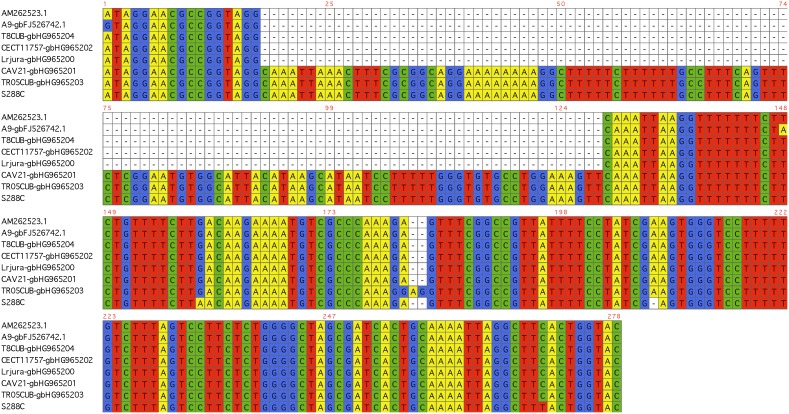
Alignment of 278 bp of ICR1 containing the deletion of 111 bp described by Fidalgo et al. [18]. The first sequences were obtained from Genbank and correspond to Spanish and an Italian flor strains [18], [48] that carry this deletion. The Spanish strain CECT11758, the Hungarian strain T8CUB and the Jura strain LRJura share the same deletion. The alleles of the Jura strain CAV21 and the Hungarian strain TR05CUB are similar to that of S288C.

The ability to develop a velum is an essential trait of flor yeast and requires high hydrophobicity at the surface of yeast cells. This trait has been related previously to polymorphisms of the *FLO11* gene [18]. Two modifications have been reported to enhance *FLO11* expression. These comprise a 111 bp deletion inside the *ICR1* non coding RNA located in the *FLO11* promoter and an increase in the size of the central part of *FLO11.* We investigated both these phenomena. First, the amplification of a fraction of *ICR1* ncRNA enabled us to detect the presence of the 111 bp deletion in the genome of 36 flor strains from the four countries, including 18 strains from the Jura 3 cluster. The cluster 2 of Spanish flor carried the wild type allele (wt) (Table 2, Figure 1). Three strains from Hungary carried both mutated and wt alleles. We sequenced the PCR amplification products of three strains originating from France (LRJura), Hungary (T8CUB), or Spain (CECT11757). Comparison of the resulting sequences with those described previously [18], [48] showed that these strains had the same deletion (Figure 6) as Spanish and Sardinian flor strains. The sequencing of this locus in two strains carrying the wild type allele, one from Jura (CAV21) and one from Hungary (TR05CUB), revealed a sequence devoid of deletion and similar to S288C.

**Table 2:** *FLO11* promoter and ORF polymorphisms, and hydrophobicity of the various flor strains.

Cluster	Strain	FLO11 diversity						Hydrophobicity			replicates
		Promoter	Flo11p length	mean per strain	mean per Cluster						
Jura 1	BAE52	del	3.2	3.2	3.7**±**0.38						
	CAH54	del	3.2	3.2							
	CAW22	del	4.2	4.2							
	CAW24	del	4.2	4.2							
	CAX22	del	3.5	3.5							
	LRJura	del	3.8	3.8				91.3**±**2.8			3
	PIA64	del	3.2	3.2				93.5**±**4.4			2
	PIN34	del	3.6	3.6				88.4**±**8.0			3
	SAC56	del	3.5; 4.2	3.9							
	XRG22	del	3.8	3.8				90.0**±**2.7			3
Jura 2	MAC51	del	3.6; 4	3.8	3.6**±**0.18			94.7**±**0.8			2
	MAD51	del	3.5	3.5							
	MAI53	del	3.5	3.5							
	XRA22	del	3.7	3.7				92.8**±**3.3			3
	XRC21	del	3; 3.7	3.35							
	SAC53	del	*ND								
Jura 3	GUF55	WT	4.7	4.7	4.7**±**0.41			90.5**±**6.6			3
	GUF51	WT	5.0	5				88.9**±**5.3			3
	CBB52	WT	4.5	4.5							
	CAV23	WT	4.7	4.7				90.7**±**4.8			2
	P5	WT	4.7	4.7				89.9**±**7.9			2
	CBD04	WT	4.8	4.8							
	GUE51	WT	4.8	4.8							
	GUG55A	WT	4.9	4.9							
	CBA13	WT	5.0	5.0							
	SAA52G	WT	6.0	6.0				94.7**±**1.8			2
	CBD05	WT	4.5; 5	4.8							
	CBE05	WT	4.5; 5	4.8							
	CAV21	WT	3.7; 4.5	4.1							
	ARC41	WT	4.5; 5	4.8				76.8**±**3.0			3
	SAA55	WT	*ND								
Jerez 2	CECT11758	del	4.5; 3.8	4.15	3.7**±**0.68			95.3**±**0.6			2
	CECT11759	del	2.7	2.7				92.4**±**2.8			3
	CECT11760	del	4.5; 3.8	4.15							
	CECT11763	del	*ND					94.1**±**0.8			3
	ET7	del	3.7	3.7				88.8**±**3.6			3
Jerez 1	480-SL	del	3.5	3.5	3.8**±**0.50			92.1**±**3.4			3
	481-SL	del	4.8; 2.7	3.75				94.0**±**3.7			3
	CECT11756	del	*ND								
	CECT11757	del	*ND								
	CECT11762	del	*ND								
	CECT12765	del	4.2	4.2							
	CECT1882	del	5; 4	4.5							
	My138	del	3.3	3.3				95.3**±**0.7			2
	My91	del	3.3	3.3							
Sardinia	1043	del	2.5		2.8**±**0.52			88.4**±**10.8			3
	FloraNero	del	3.4					91.8**±**5.4			3
	M25	del	2.5					35.4**±**5.5			3
Hungary	T19CUB	del	2.0		3.0**±**0.62						
	T8CUB	del	3.3								
	TS12CUB	del	2.4					93.9**±**1.2			3
	TA12CUB	WT del	3.5					94.8**±**0.9			3
	TP32CUB	WT del	3.0					95.0**±**2.0			3
	TR05CUB	WT del	3.5								
Spanish Flor 2	G1	WT	2.2	2.2	2.2			10.0**±**7.8			3
Wine Cluster	MAA52	WT	2.4	2.4	2.9**±**0.81						
	MTF2-K1	WT	2.4	2.4				8.7**±**7.0			3
	RM11	WT	3.8	3.8							
Lab	S288C	WT	3.2					16.7**±**5.8			3

del: presence of the deletion in ICR1, WT: Wild type allele. The size of the core region of Flo11p alleles is given, as well as the mean size per strain. The mean size of Flo11p per cluster is given with standard variation. Hydrophobicity was measured according to Ishigami et al. [17], and is expressed as mean of replicates +/− standard deviation. The number of replicates is given in the last column.

*ND: could not be amplified.

We amplified the core region of Flo11p for 59 strains and obtained DNA fragments for 53 strains, with sizes varying from 2.5 to 6 kb. We did not obtain amplification for four Spanish strains and two Jura strains. The mean size for wine strains was 2.9 kb, similar to Hungarian flor strains at 3.0 kb. The core region of Flo11p was longer in other flor groups, including Jura 1 and 2 at 3.6 kb and Jerez 1 and 2 at 3.7 kb.Jura 3 cluster strains had the longest Flo11p core region (4.8 kb). We obtained a mean value of 2.8 kb for three Sardinian strains. The size of the variable central core of *FLO11* was evaluated previously [48] with a different primer pair for Sardinian strains. These primers were closer to the central variable core of Flo11p than those used in our study; therefore, we recalculated the mean size obtained with our primers as 3.4 kb for the 22 genotyped strains, which is similar to most flor yeast groups, and lower than the size measured for Jura 3 cluster strains. This indicates that most flor strains contain a deletion in *ICR1* and have a core region of Flo11p that is longer than that of wine strains. Strains of the Jura 3 cluster have a particular combination of *FLO11* alleles with a full length *ICR1* and a very long core region.

We measured cell hydrophobicity and velum formation to examine the effect of *FLO11* polymorphism on phenotype (Table 2). We measured the hydrophobicity of 28 strains, and found highly significant differences both between strains (*p*. value of a one factor ANOVA <2 e^−16^) and groups (*p*. value of a Kruskal Walis Test = 4.63 e^−5^). As expected, the flor and the “non-flor” group, including K1 wine strains, the Spanish flor 2 G1 strain and the reference strain S288C, showed the largest differences in hydrophobicity. We also found significant differences between the three Sardinian strains and the “non-flor” group (*p*. value of Kruskal Walis Test = 0.00034) but not with other flor yeast. The hydrophobicity of Jura 3 cluster strains was similar to that of other flor groups.

We assessed the ability of 29 strains to produce a velum by cultivating them on Fornachon’s media. All strains of clusters Jura 1, Jura 2 and Jerez 2 produced a velum (Table 3). The growth of strains of clusters Jerez 1, Jura 3, Sardinia, and Hungary was variable. Five out of six strains from the Jura 3 cluster, and several strains from Hungary (TR05CUB, TP32CUB, TA12CUB, TS12CUB) either produced a thin velum or no velum at all. Wine strains and the two atypical Spanish flor strains were unable to develop a velum in this media. We previously correlated velum thickness and color in Jura flor yeast with genetic group assessed by interdelta typing [8]. Almost all of the strains analyzed in this prior study were genotyped; therefore, we were able to evaluate the correlation between genetic structure revealed by microsatellite typing and the ability to produce a velum for these strains. The correlation between microsatellite structure and the production of thin velum in Jura 3 cluster strains (*p*. value of ***χ*** ^2^ test<6.7 e^−10^ and 1.0 e^−07^ for color and thickness respectively, for 55 strains) was substantially higher than that we obtained previously between velum production and delta clusters (*p*. value of ***χ*** ^2^ test<0.0007 and 0.0076 for color and thickness respectively) [8].

**Table 3:** Growth of the various strains on Fornachon’s media. Intensity of velum formation is scored from 0 (no velum) to 4 (thick velum).

Microsatellite	Duration	Incubation (days)				
**cluster**	Strain	2	4	6	8	10
**Jura 1**	BAE52	2	4	4	4	
	LR	1	4	4	4	
	PIN34		2	3	3	fell
	MAC51		4	4	4	4
**Jura 2**	GUF55		1	1	1	0
	MAD 51		4	4	4	fell
	MAI53	4	4	4	4	
**Jura 3**	ARC41	0	0	0	0	0
	CAV21	0	0	0	0	0
	CBD04		0	0	0	0
	GUG55	0	4	4	4	4
	GUE 51		1	0	0	1
	P5	0	0	1	1	0
**Jerez 1**	480 SL		0	0	0	0
	481 SL		4	4	4	0
	MY138	0	3	3	2	
**Jerez 2**	CECT11758	0	0	1	1	
	CECT11763	0	4	4	4	
	ET7	0	0	1	1	
**Sardinia**	1043	0	0	0	0	0
	Flora Nero	0	1	3	3	3
	M25	0	0	0	0	0
**Hungary**	T19CUB	0	3	3	3	4
	T8CUB	2	4	4	4	4
	TR05CUB	0	0	0	0	0
	TP32CUB	0	1	0	1	0
	TA12CUB	0	0	0	1	1
	TS12CUB	0	0	0	0	0
**Spanish Flor 2**	G1	0	0	0	0	0
**Lab**	S288C	0	0	0	0	0

## Discussion

Flor strains are found in several countries in Europe; however, until now no global approaches had been undertaken to compare strains from various vineyards. We showed previously that Jura flor strains carry a specific allele of *ITS1*, which differs from that characterized in Spanish strains [5], [8], suggesting the existence of separate populations. In addition, a previous study on Spanish flor yeast revealed that flor yeast are genetically isolated from wine fermentation yeast during the aging process [7], suggesting that flor strains represent a separate family of *Saccharomyces cerevisiae*.

In this study, we used microsatellite typing, InStruct clustering and population analysis to reveal for the first time that most flor strains share the same unique origin. Lebanese and Spanish strains showed the most basal position within the population structure; therefore, it is difficult to infer the origin of flor yeast. Interestingly, a flor yeast population was recently characterized in Georgian aged wines produced by the “Kakhetian” method [49]. Nonetheless, it is still possible that all flor strains have a Mesopotamian origin because wine making is an ancient process in Georgia and this country is close to origin of vine domestication. However, the comparison of a larger number of strains is necessary. The position of the Jura strains at the end of the branch of the population Fst network suggests that Jura flor strains have a lower diversity than Spanish or Italian populations, indicating that this vineyard received strains from other vineyards. This can be seen also from InStruct clustering: Spanish flor strains are mostly mosaics of two origins (at K = 9), with a third origin for some strains, whereas half of Jura strain are associated with only one cluster. Hungarian strains are closely related to the Jura population as shown by the network and InStruct output. The second Spanish flor cluster is associated with some rum strains as seen from the individual tree and these strains share ancestry according the InStruct output (three individuals at the right of the Spanish flor strain cluster).

Wine is a much harsher environment than must for yeast cells during flor aging. During alcoholic fermentation, yeast cells metabolize almost all fermentable sugars and assimilate most nitrogen sources (except proline) and vitamins. As a result, wine contains a high concentration of alcohol (starting from 13% v/v in Jura, and 14–15% in Sardinia and Spain) and a low nitrogen and vitamin content. In addition, yeast cells have an aerobic biofilm lifestyle, and use glycerol and ethanol as carbon sources. Many experiments have shown how yeast are able to adapt to particular environmental conditions [50]–[53] through various adaptive genetic changes [54], [55]. The intense stressful conditions of flor aging to which flor yeast cell are subjected for years of growth may drive such adaptation.

Aneuploidy is a mechanism that fuels adaptation to environmental changes [15], [50]. Comparative Genome Hybridization on array (aCGH) has enabled the exploration of gene copy number variations. This technique revealed that wine yeast share a genomic signature [44], [45]. Aneuploidies have also been detected in the genome of flor yeast [12], [13] and proposed as a motor for adaptation. In addition, recent studies show that gene duplication or loss is specific of certain lineages, suggesting that it can offer a shortcut to evolutionary adaptation [47]. A recent aCGH study examined the genetic constitution of strains of different origins including one flor yeast [56]. The array technology and data processing method used in this study differs from that used here; nonetheless, findings for the triploid flor strain GB-FlorC are similar between the two studies: Ibanez et al. found that the genes *YKL221W/MCH2* and *YKL222C* were among 81 genes showing a log ratio greater than 0.5 with S288C used as a reference strain. The *YHR215W/PHO12* gene, which is amplified in LRJura and My138, was also included in this list. In addition, for the flor strain GB-FlorC, half of all genes with a log ratio lower than −0.7 were also included in the list of genes with a low hybridization signal of flor strains analyzed here. Our investigation has two limits: (1) we cannot exclude the possibility that some genes were missed by our data analysis; and (2) our findings are limited to comparison with the S288C genome; therefore, we did not take into account genes detected specifically in wine yeast such as A, B, and C regions identified in EC1118 [57]. Our aCGH analysis and that of Ibanez et al. [56] do not support the view that many gene amplification events must occur to enable the adaptation of yeast to the flor aging environment. We hypothesize that the substantial differences observed previously [12] originate partially from differences in ploidy between the two Spanish strains and that these differences are a specific feature of this pair of strains as opposed to a general adaptive pattern. However, recent observations show that aneuploidies appear in the first steps of adaptation [58], but are subsequently replaced by other mutations, probably because of the cost of aneuploidy. Pulsed field gel electrophoresis to examine the genetic variability of wine and flor yeast has also revealed the importance of aneuploidy in yeast adaptation. It is possible that the numerous variations observed with this technique result from translocations, which can also generate new phenotypes as shown previously for *SSU1* [59], or from specific gene clusters such as those detected in EC1118 [57]. Such clusters may be inserted at different loci with a variable number of copies [60], [61]. However, we successfully identified amplified genes shared by flor yeasts, including two genes: *YKL221W/MCH2* and *YKL222C. MCH2* is a putative monocarboxylic acid transporter with homology with mammalian transporters, although its involvement in monocarboxylic acid transport has not been shown experimentally [62]. Nonetheless, a recent study showed that this gene is important for yeast survival during the second phase of alcoholic fermentation (during alcohol accumulation) [63]. In addition, Zara et al. found that succinic, lactic and acetic acids could not provide consistent growth as a sole carbon source under aging conditions [64]. The role of *YKL222C* is also unknown; however, a recent overexpression screen to identify genes involved in endocytic trafficking, suggested a role for Ykl222cp in the early endosome or during endocytosis [65].

Several genomic regions showed a low hybridization signal indicating that these regions are missing or contain variations hampering hybridization. One of the most puzzling aspects was the location of most of these events in subtelomeric regions, which was observed previously by other groups [44], [45], [56]. The low number of copies of several genes in contrast with the amplification of other genes suggests translocation between subtelomeric regions. Indeed, several translocations have been shown to play a key role in the adaptation of yeast to selective pressure [15], especially in the response of wine yeast to sulfite exposure [59], [66]. Unfortunately, we were unable to detect directly translocation events from our data. In addition, linkage analysis has revealed that these regions play a key role in defining individual quantitative variation and thus in the adaptation of natural populations [67].

Polymorphism of *FLO11* is also a key feature of flor strains. The global hydrophobicity of flor cells is determined by the level of *FLO11* expression and Flo11p length [18], [48]. Our results are in line with these findings we correlated flor yeast population structure data with *FLO11* polymorphisms. We detected the 111 bp deletion, first observed by Fidalgo [18], in Spanish, Italian, Hungarian, and French strains, suggesting that it is extremely old. Only two Hungarian strains were heterozygote at this locus indicating that this deletion has probably been selected for by most flor strains. As a result, the wild type allele has nearly disappeared from flor strains, except in particular groups such as the Jura 3 cluster. Thus, various adaptive strategies enabling yeast cells to overcome the stressful conditions of flor aging co-exist, similar to what has been observed in experiments of adaptive evolution [54].

In conclusion, our results reveal that flor yeast are a unique family. Flor strains are mainly diploids, with some polyploid Spanish strains. We detected a shared pattern of amplification for two genes in four out of six flor strains (*MCH2* and *YKL222w*) and identified genomic regions with low hybridization to probes based on the S288C genome. These regions were mainly located in subtelomeric regions, which may be associated with a high level of divergence and thus explain adaptation to flor aging. In addition, *FLO11* polymorphisms suggest that several alternative strategies can lead to adaptation to flor aging. Further investigation is required to unravel the mechanisms of flor yeast adaptation, in particular studies involving genome sequencing.

## Supporting Information

Figure S1 **Evolution and variability of Deviance Information Criteria for different values of K.**
(PDF)

Figure S2 **Clustering of flor strains with InStruct population structure inference software for K = 3 populations.** Each color corresponds to one inferred ancestral group. The proportion of each color gives the proportion of the corresponding ancestral genome in the genome of each strain. The name of the isolated population is shown at the top of each cluster.
(TIFF)

Figure S3 **Karyoscope obtained with DNAcopy, showing variations in hybridization signal along the chromosome for 8 other strains: 5 flor strains P3, FloraNero, LRJura, CECT11758, TA12CUB, 2 wine strains Eg25, V5, and aneuploidy hybrid** Saccharomyces cerevisiae*S.kudriavzevii **Eg8.** Chromosomes are colored in blue (uneven numbers) or dark blue (even numbers). Mean segment level estimated by DNAcopy is shown as a red line. The red arrow indicates the *YKL221W/MCH2* and *YKL222C* region, and the orange arrow indicates the *PHO12* and *IMD2* region.
(EPS)

Table S1 **Origin of the different strains analyzed in this study.**
(XLSX)

Table S2 **List of genes showing variation in hybridization between tested strains and the reference strain S288C.**
(XLSX)

Table S3 **Comparison of the various gene lists obtained according to hybridization signal.**
(XLSX)
