# Supplementary material for: Population Structure and Comparative Genome Hybridization of European Flor Yeast Reveal a Unique Group of Saccharomyces cerevisiae Strains with Few Gene Duplications in Their Genome
Source: PLoS One. 2014 Oct 1;9(10):e108089. doi: 10.1371/journal.pone.0108089 (PMC4182726; doi:10.1371/journal.pone.0108089)

Figure S1: A. Evolution of DIC with K (obtained for 10 runs)
B. Evolution of posterior variance for the different values of K

A.

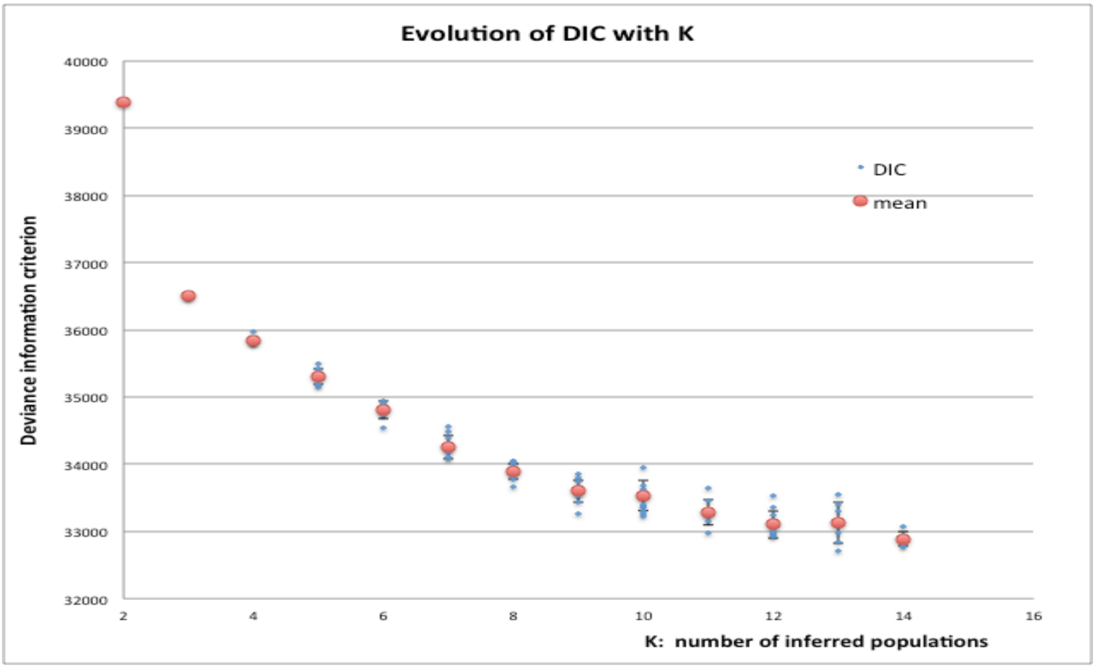

B.

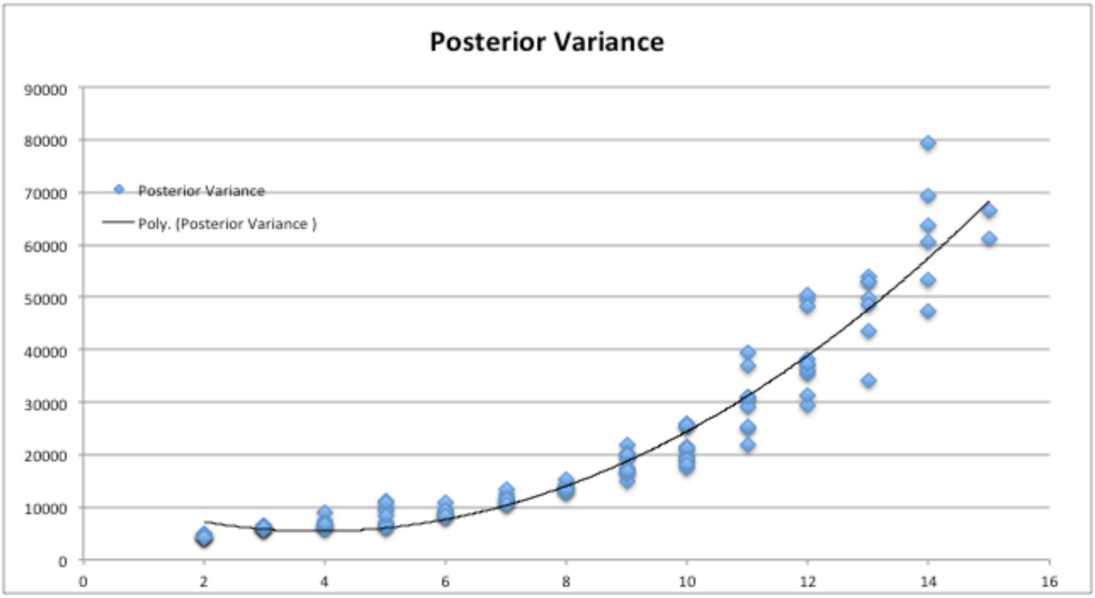

Supplement: Figure S1 — Evolution and variability of Deviance Information Criteria for different values of K. (PDF) [file pone.0108089.s001.pdf]
