# Supplementary figures and images for: Population Structure and Comparative Genome Hybridization of European Flor Yeast Reveal a Unique Group of Saccharomyces cerevisiae Strains with Few Gene Duplications in Their Genome
Source: PLoS One. 2014 Oct 1;9(10):e108089. doi: 10.1371/journal.pone.0108089 (PMC4182726; doi:10.1371/journal.pone.0108089)

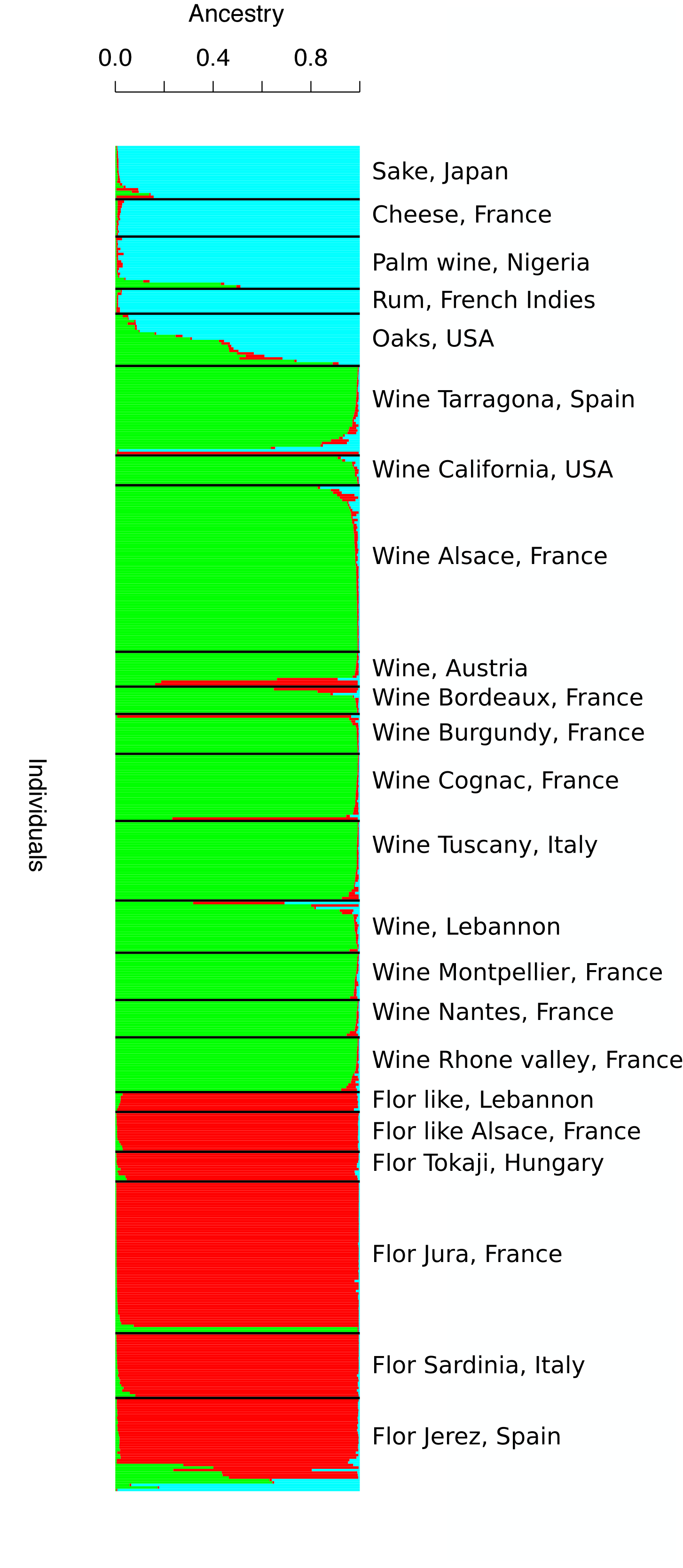

Supplement: Figure S2 — Clustering of flor strains with InStruct population structure inference software for K = 3 populations. Each color corresponds to one inferred ancestral group. The proportion of each color gives the proportion of the corresponding ancestral genome in the genome of each strain. The name of the isolated population is shown at the top of each cluster. (TIFF) [file pone.0108089.s002.tiff]
